# DNA metabarcoding using nrITS2 provides highly qualitative and quantitative results for airborne pollen monitoring

**DOI:** 10.1016/j.scitotenv.2021.150468

**Published:** 2022-02-01

**Authors:** Marcel Polling, Melati Sin, Letty A. de Weger, Arjen G.C.L. Speksnijder, Mieke J.F. Koenders, Hugo de Boer, Barbara Gravendeel

**Affiliations:** a Naturalis Biodiversity Center, Leiden, the Netherlands; b Natural History Museum, University of Oslo, Norway; c Department of Pulmonology, Leiden University Medical Center, Leiden, the Netherlands; d Leiden University of Applied Sciences, Leiden, the Netherlands; e Clinical Chemistry, Elkerliek Hospital, Helmond, the Netherlands; f Radboud Institute for Biological and Environmental Sciences, Nijmegen, the Netherlands

**Keywords:** Aerobiology, Airborne pollen, DNA metabarcoding, nrITS2, Quantification, *trn*L P6 loop

## Abstract

Airborne pollen monitoring is of global socio-economic importance as it provides information on presence and prevalence of allergenic pollen in ambient air. Traditionally, this task has been performed by microscopic investigation, but novel techniques are being developed to automate this process. Among these, DNA metabarcoding has the highest potential of increasing the taxonomic resolution, but uncertainty exists about whether the results can be used to quantify pollen abundance. In this study, it is shown that DNA metabarcoding using *trn*L and nrITS2 provides highly improved taxonomic resolution for pollen from aerobiological samples from the Netherlands. A total of 168 species from 143 genera and 56 plant families were detected, while using a microscope only 23 genera and 22 plant families were identified. NrITS2 produced almost double the number of OTUs and a much higher percentage of identifications to species level (80.1%) than *trn*L (27.6%). Furthermore, regressing relative read abundances against the relative abundances of microscopically obtained pollen concentrations showed a better correlation for nrITS2 (R^2^ = 0.821) than for *trn*L (R^2^ = 0.620). Using three target taxa commonly encountered in early spring and fall in the Netherlands (*Alnus* sp., Cupressaceae/Taxaceae and Urticaceae) the nrITS2 results showed that all three taxa were dominated by one or two species (*Alnus glutinosa/incana*, *Taxus baccata* and *Urtica dioica*). Highly allergenic as well as artificial hybrid species were found using nrITS2 that could not be identified using *trn*L or microscopic investigation (*Alnus* × *spaethii*, *Cupressus arizonica*, *Parietaria* spp.). Furthermore, perMANOVA analysis indicated spatiotemporal patterns in airborne pollen trends that could be more clearly distinguished for all taxa using nrITS2 rather than *trn*L. All results indicate that nrITS2 should be the preferred marker of choice for molecular airborne pollen monitoring.

## Highlights

• DNA successfully extracted from microscopic pollen slides and Burkard-collected tapes.
• nrITS2 and *trn*L DNA metabarcoding improves taxonomic resolution of airborne pollen.
• Relative read abundance nrITS2 shows higher correlation with pollen counts than *trn*L.
• Finer scale spatiotemporal patterns in pollen trends detected using nrITS2.
• Detection of artificial hybrid (*Alnus* × *spaethii*) that significantly prolongs hay fever season

## Graphical abstract

**Figure:**
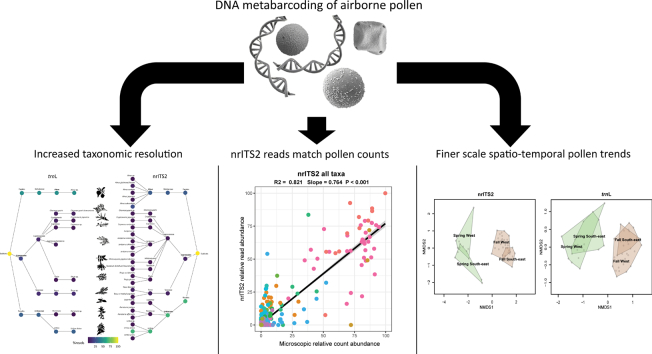

## 1 Introduction

With hay fever incidence on the rise in the 21st century, monitoring of pollen in ambient air is of high socio-economic relevance to both health care and research (Anderegg et al., 2021; Suanno et al., 2021). The diversity of pollen in ambient air is typically monitored using pollen traps and microscopic identification. This information is important for hay fever patients, but it is a time-consuming process that requires highly trained specialists. Automating pollen counting and identification using new technologies (Dunker et al., 2021; Sauvageat et al., 2020) or by using deep learning algorithms on pollen images (Olsson et al., 2021; Schaefer et al., 2021; Sevillano et al., 2020) has been shown to increase speed and accuracy. However, these methods do not generally improve the taxonomic resolution of pollen identifications. Neural networks have in some cases been shown to increase taxonomic resolution for pollen that cannot be separated by specialists by their morphology (Polling et al., 2021; Romero et al., 2020). This technique, however, requires an extensively trained network with varied pollen images and high-resolution microscopes, and does not work for all pollen types. Since many important allergenic plant families like Poaceae, Urticaceae and Cupressaceae/Taxaceae are stenopalynous (i.e. produce morphologically identical pollen), much information on the relative abundance and spatial patterns of individual species is lost (Erdtman, 1986; Kurmann, 1994). This information is important as different species may possess different allergenic profiles and ecological preferences (e.g. D'Amato et al., 2007). Moreover, it is currently impossible to obtain information on airborne pollen from many cultivated and exotic species versus native plant species.

As an alternative to morphological pollen identification, DNA metabarcoding has been shown to provide increased taxonomic resolution and it has been used successfully on bee-collected pollen (Bänsch et al., 2020; Elliott et al., 2021; Gous et al., 2021; Richardson et al., 2019) as well as airborne pollen (Banchi et al., 2020; Brennan et al., 2019; Campbell et al., 2020; Kraaijeveld et al., 2015; Uetake et al., 2021). For grasses (Poaceae) for example, a recent study has shown that pollen of a small subset of all species present in the UK is likely to have a disproportionate influence on human health (Rowney et al., 2021). However, such highly detailed information is not yet available for other plant families.

Increasingly, studies are demonstrating that the relative abundance of metabarcoding read counts shows a good correlation with relative abundances of microscopically obtained pollen concentrations (e.g., Kraaijeveld et al., 2015; Richardson et al., 2021; Richardson et al., 2019), although this correlation may depend on both the species studied as well as the other species present in the mixture (Bänsch et al., 2020; Bell et al., 2019). Furthermore, since pollen from different species possesses different copy numbers of plastid and nuclear DNA, this correlation may be highly dependent on the marker choice (Bell et al., 2016a; Rogers and Bendich, 1987). Commonly used DNA marker regions in pollen metabarcoding include plastid *rbc*L and *trn*L as well as the nuclear ribosomal Internal Transcribed Spacer (nrITS) regions ITS1 and ITS2. For complex aerobiological samples containing pollen from various species as well as fungal spores, bacteria and viruses, the correlation between microscopic pollen concentrations and DNA reads has been found to be relatively low using the *rbc*L plastid marker (Campbell et al., 2020; Uetake et al., 2021). While *trn*L has shown promising results in quantifying pollen (Kraaijeveld et al., 2015), it has not yet been tested on a large dataset and nrITS2 has not been sufficiently tested for aerobiological samples.

In this study we first test whether DNA metabarcoding using plastid *trn*L and nuclear ribosomal ITS2 loci can be used to increase taxonomic resolution of airborne pollen identifications. Pollen samples were collected from two pollen monitoring stations in the Netherlands, with a focus on three commonly encountered pollen types in the Netherlands in early spring and fall (*Alnus* sp., Cupressaceae/Taxaceae and Urticaceae). These three taxonomic groups were selected because we identified a high need for improved taxonomic accuracy for these groups as this is currently limited with traditional microscopy. For these three groups, exotic allergenic species are currently establishing themselves as part of the wild flora in the Netherlands and elsewhere in the world. Apart from that, within these taxa we identified several highly allergenic species (e.g. *Parietaria* spp.) which currently cannot be identified. The alders (*Alnus*) can be identified to the genus level under a microscope, while nettles (Urticaceae) can only be recognized to the family level. Cypress (Cupressaceae) pollen cannot be distinguished from pollen of the yew family (Taxaceae). Using the three target taxa, the quantitative performances of the two DNA markers are compared to microscopic pollen concentrations. The quantitative results are used to visualize trends in species that could hitherto not be distinguished using traditional methods. We also investigate whether DNA metabarcoding shows significant differences between the two pollen monitoring sites in early spring and fall.

## 2 Material and methods

### 2.1 Material

**Fig. 1:**
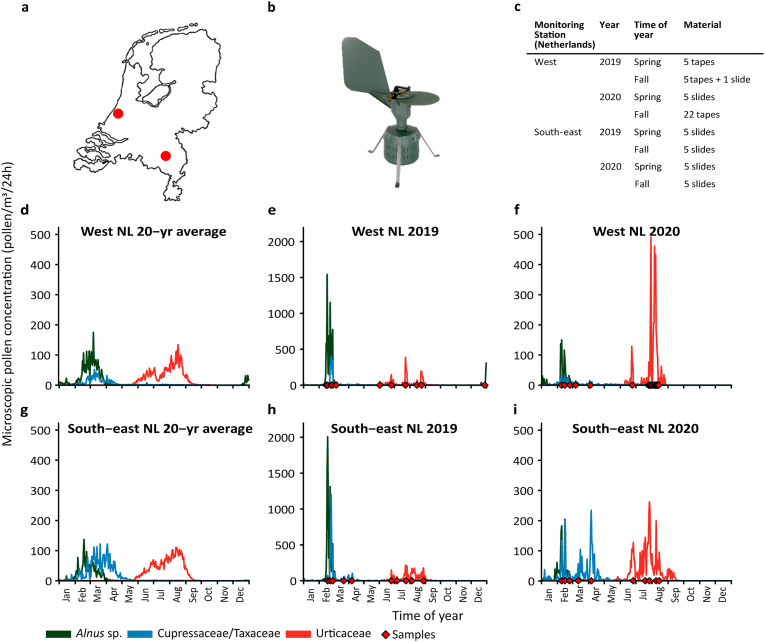
Pollen collection in the Netherlands a) locations of pollen monitoring sites, the West (Leiden) and South-east of the Netherlands (Helmond) b) Hirst-type Burkard pollen sampler c) sample selection of Melinex tapes and microscopic slides with mounted tapes d,g) 20-year average pollen concentrations of *Alnus*, Cupressaceae and Urticaceae at both pollen monitoring stations e,h) 2019 pollen concentrations of the three target taxa and f,i) 2020 pollen concentrations. Sampling dates are shown with red diamonds on the x-axis. Note scale change for figure e and h. NL = the Netherlands, yr = year. (For interpretation of the references to colour in this figure legend, the reader is referred to the web version of this article.)

Samples used in this study were collected in 2019 and 2020 at two airborne pollen monitoring stations in the Netherlands, including the Leiden University Medical Center (LUMC), Leiden, West of the Netherlands and Elkerliek Hospital in Helmond, South-east of the Netherlands (Fig. 1a). These stations routinely collect airborne pollen from ambient air for allergenic pollen monitoring using a Burkard spore trap (Burkard Manufacturing, Rickmansworh, UK) (Fig. 1b). This device has been placed on top of the roof of LUMC since 1969 and the Elkerliek Hospital since 1975. The Burkard trap continuously sucks in air using a vacuum pump and impacts any particles >3.7 μm on a Melinex adhesive tape mounted on a drum that rotates behind the inlet in 7 days. Since the drum rotates at a constant speed, a given section of tape corresponds to a known length of time. This tape is cut into seven pieces of 48 mm, each corresponding to 24 h, from which a microscopic slide is prepared. Pollen slides are made by placing the Melinex tapes on a microscopic glass slide and mounted using a glycerin:water:gelatin (7:6:1) solution with 2% phenol and stained with Safranin (0.002% w/v). A cover glass is placed over the tape which is sealed with nail polish.

This study focuses on three taxonomic groups in particular (*Alnus* sp., Cupressaceae/Taxaceae and Urticaceae), and samples with high pollen concentrations in these taxa were selected. As has been shown before, samples with too few pollen grains may not yield sufficient DNA for downstream analysis (Baksay et al., 2020). Samples were selected from either late winter – early spring (February to May) for *Alnus* and Cupressaceae/Taxaceae or summer – early fall for the Urticaceae (Fig. 1e-f, h-i). When referring to these time periods from now on in this manuscript the terms ‘spring’ and ‘fall’ will be used, and ‘Cupressaceae’ is used from now on when referring to Cupressaceae/Taxaceae. The 20-year pollen concentration averages from the two pollen monitoring sites show broadly similar patterns for *Alnus* sp., although a peak in late December – early January is only observed in the West of the Netherlands (Fig. 1d). This is expected to be caused by the flowering of non-native artificial hybrid *Alnus* × *spaethii* that is commonly planted along city streets because of its drought resistance (Jablonski, 2018). According to presence and abundance data of Spaeth's Alder accumulated on the Dutch citizen science platform waarneming.nl, spontaneous seedlings of this cultivar have been detected in more and more parts of the Netherlands over the past decade. Cupressaceae are notably more abundant in the South-east of the Netherlands, while Urticaceae show a similar ‘twin-peak’ abundance pattern (early July and late August; Fig. 1d, g). For metabarcoding analysis in this study, we had access to 20 tapes mounted on microscopic slides from the South-east of the Netherlands. From the West of the Netherlands we obtained 6 mounted tapes as well as 32 unmounted tapes (Fig. 1c). The unmounted tapes from the West of the Netherlands were obtained daily from a second (backup) Burkard device placed two meters away from the first. Mounted tapes were stained with safranin and preserved in glycerol, both of which were identified as potential inhibitors for DNA amplification. Daily pollen concentrations (pollen/m^3^/24h) were calculated after counting pollen on microscopic slides in three longitudinal bands at 40× magnification under the microscope (Galán et al., 2017).

### 2.2 Study area

The pollen monitoring station in the West of the Netherlands is located on the roof of the LUMC, just West of the city center of Leiden, within a larger urbanized area of the Netherlands termed the ‘Randstad’. Dunes are located approximately 7 km to the West of Leiden, with tulip and lily bulb fields and intensively managed pastures in between. North-east and South of Leiden there are several small lakes and pastures, while closer to the monitoring site the setting is mostly urban with a railway station and parking lots. Some parks and other green areas are present, mostly dominated by *Alnus*, *Betula* and *Populus* spp. (Spieksma et al., 2003). The pollen monitoring station on the roof of the Elkerliek hospital in the East of the Netherlands is located just North-west of Helmond, with intensively managed farmland and forest in the surrounding area. A patch of recreational forest dominated by *Quercus* and *Fagus* is located just West of the hospital (Spieksma et al., 2003). Private gardens and public parks are present in the direct surrounding of both monitoring stations, as well as many types of cultivated trees along the streets. For more detailed information on the pollen monitoring sites, please refer to De Weger et al. (2021).

### 2.3 Methods

#### 2.3.1 DNA extraction and amplification

All the next steps were performed in a flow cabinet in a dedicated DNA clean room laboratory of Naturalis Biodiversity Center (Leiden, the Netherlands). To extract the Melinex tape from the microscopic slide, the outside surface of the slide was cleaned sequentially with 70% EtOH and 1:100 Chlorine solution to remove potential contamination. Slides were then placed on a heating plate for several seconds to dissolve the nail polish that was used to seal the cover glass, and the cover glass was carefully lifted with UV-cleaned tweezers to remove the tape. The above-mentioned steps were part of a process of trial and error, as to our knowledge no previous studies have attempted this before. From here, the procedure was the same as that used for the tape directly obtained from the backup Burkard sampler, and mostly following methodologies from Kraaijeveld et al. (2015). Half of the Melinex tape was cut horizontally for DNA analysis while the other half was preserved for future analysis. The tape for DNA extraction was cut in small pieces and placed in a 2 ml tube. Prior to DNA extraction, pollen cell walls were disrupted using the pollen lysis protocol described in Kraaijeveld et al. (2015), adjusted by using four 2.3 mm stainless steel and ten 0.5 mm glass beads, and disrupting the pollen in a Retsch Mixer Mill MM 400 for 3 × 2 min at 30 Hz. After bead beating, 100 μl of 5% sodium dodecyl sulfate (SDS) was added to the samples and these were incubated at 65 °C for 30 min. DNA was extracted using the QIAamp DNA Mini kit according to the manufacturers’ protocol (Qiagen). Extraction blanks (Melinex tape without pollen) were included in each round of extractions and these were pooled per three during the PCR step resulting in two sets of extraction blanks in the final dataset.

A two-step PCR protocol was used to create a dual index amplicon library, using the *trn*L primers *g* and *h* to amplify the chloroplast *trn*L intron P6 loop (Taberlet et al., 2007) and the plant-specific primers ITS-p3 (Cheng et al., 2016) and ITS4 (White et al., 1990) to amplify nuclear ribosomal Internal Transcribed Spacer region nrITS2. We used three PCR replicates per sample (giving each a unique tag combination). All extraction blanks, PCR negative blanks (seven) and positive controls (two; pollen from non-native *Citrus japonica*) were included in both rounds of PCRs and sequencing. First round PCRs were carried out in 25 μl reactions containing 14.75 μl nuclease-free ultrapure water, 1× Phire Green Reaction Buffer (Thermo Scientific), 1.0 μl of each 10 mM primer, 0.5 μl of 1.25 mM dNTP's, 0.5 μl Phire Hotstart II DNA Polymerase and 1.0 μl of sample DNA extract. This mixture was denatured at 98 °C for 30 s, followed by 35 cycles including 5 s at 98 °C, 5 s annealing at 55 °C for *trn*L or 58 °C for nrITS2, extension at 72 °C for 15 s and a final extension at 72 °C for 5 min. PCR success was checked on an agarose gel. All PCR products were cleaned using one-sided size selection with Agencourt AMPure XP beads (Beckman Coulter), at a 1:0.9 (nrITS2) or 1:1 ratio (*trn*L).

To add individual P5 and P7 Illumina labels to all samples (Nextera XT Index Kit; Illumina, San Diego, CA, USA), a second round of PCRs was performed in a final volume of 20 μl using 3.0 μl of the cleaned PCR product from the first round, 5.0 μl ultrapure water, 10.0 μl KAPA HiFi HotStart ReadyMix (KAPA Biosystems, Boston, Massachusetts, USA) and 0.5 μM of each Illumina label. The PCR program included an initial denaturation at 95 °C for 3 min followed by eight cycles of 20 s at 98 °C, 30 s at 55 °C and 30 s at 72 °C, followed by a final extension at 72 °C for 5 min. The resulting PCR products were pooled into two pools based on amplicon length: a pool containing the shorter *trn*L fragments and one containing the longer nrITS2 fragments. For each marker a library was constructed by equimolar pooling of the PCR products after measuring amplicon concentrations on a QIAXcel (Qiagen). The pools were purified using Agencourt AMPure XP beads (Beckman Coulter), with a 1:0.9 ratio for nrITS2 and 1:1 for *trn*L, and quantified using an Agilent 2100 Bioanalyzer DNA High sensitivity chip (Agilent Technologies, Santa Clara, CA, USA). The pools were sequenced in separate runs on an Illumina MiSeq (v3 Kit, 2x300 paired-end) at Baseclear (Leiden, the Netherlands). Raw sequence data is available at ENA project nr PRJEB45538.

#### 2.3.2 Bioinformatics and filtering

The sequences were analysed on a custom pipeline on the OpenStack environment of Naturalis Biodiversity Center through a Galaxy instance (Afgan et al., 2018). Raw sequences were merged using FLASH v1.2.11 (Magoc and Salzberg, 2011) with a minimum overlap of 10 bp and maximum mismatch ratio of 0.25, discarding all non-merged reads. Primers were trimmed from both ends of the merged reads using Cutadapt v2.8 (Martin, 2011). Any reads without both primers present (allowing a maximum mismatch of 0.2) or shorter than 8 bp (*trn*L) or 150 bp (nrITS2) were discarded. Sequences were dereplicated and sorted by size in VSEARCH v2.14.2 (Rognes et al., 2016) and clustered into “zero-noise” Operational Taxonomic Units (OTUs) using the *unoise3* algorithm from USEARCH v11.0.667 (Edgar, 2016) with default settings and a minimum abundance of 10 reads before clustering, removing singletons and potential chimeras. The resulting OTU sequences were compared to two taxonomic reference libraries for both markers. In order to avoid false BLAST hits, custom reference databases were constructed for both markers consisting of all native and introduced plants from the Netherlands (obtained from https://www.verspreidingsatlas.nl/soortenlijst/vaatplanten and including recent arrivals from Denters (2020)). This list was further supplemented with a list of all cultured plants in the Netherlands, obtained from the ‘Standard list of Dutch culture plants 2020’ (Marco Hoffman, pers. comm.) resulting in a list of 19,561 green plant taxa. All available *trn*L and nrITS2 sequences belonging to species on this list were downloaded from NCBI GenBank on 21 April 2021, resulting in a reference library of taxa occurring in the Netherlands consisting of 8391 sequences for *trn*L and 10,015 for nrITS2. To mitigate erroneous or missing taxonomic assignment due to references potentially missing in the Dutch custom databases, a second reference library was constructed for both markers, consisting of worldwide *trn*L and nrITS2 plant sequences, downloaded from NCBI GenBank on 21 April 2021. Priority was given to the local database and if multiple blast hits were found with the same maximum BIT-score, the lowest common ancestor of these hits was chosen. A minimum of 97% identity was used for species level identification, 90% for genus and 80% for family (following e.g. Ghorbani et al., 2017). For *trn*L only sequences with a 100% cover were accepted, while this value was 90% for nrITS2 to account for incomplete reference sequences in the database (partial ITS2 records). Finally, OTUs with the same taxonomic assignment were aggregated.

The resulting sequences were further filtered in R (version 3.5.2; Team, R. Core, 2013) to remove a) OTUs that were more abundant in negative or extraction blanks than in samples, b) sequences present with <10 reads per PCR repeat, c) potential leakage, using a custom R script to determine the filtering threshold that would result in removal of all reads from negative controls (0.0035% (nrITS2) and 0.05% (*trn*L) of each sequence read count per sample) d) PCR repeats with fewer than 3000 reads, e) OTUs from fungi, bryophytes or green algae, f) any OTUs that were present in only one of the three PCR repeats (see Table S1 for all filtering steps and read counts). Several samples (12 for nrITS2 and one for *trn*L) had only one PCR replicate left after these filtering steps. Since these samples could not be cleaned using the minimum threshold of two PCR repeats, they were carefully checked for potential contaminations.

Several suspicious OTUs of potential food contaminants still remained in both datasets after these filtering steps. The microscopic slides that we analysed were not made with DNA metabarcoding in mind, and no particular precautions were taken to avoid contamination. This may explain the presence of, e.g., *Arachis hypogaea* (peanut), *Glycine* *max* (soj), *Ananas comosus* (pineapple) and *Persea americana* (avocado) in the *trn*L results (Fig. S2). However, we also found DNA from *Solanum lycopersicum* (tomato), *Secale cereale* (rye), *Pisum sativum* (pea) and *Phaseolus vulgaris* (common bean; among others) that grow naturally and are commonly cultivated in the Netherlands. However, since DNA from many of these species was found in samples from both spring and fall, they were conservatively assumed to be derived from contamination. This approach was adopted across all OTUs, and OTUs from potential food contamination were removed (see Fig. S1-2 for all removed taxa).

#### 2.3.3 Data analysis

The reads from the remaining replicates were averaged and converted to relative read abundances (RRA) using the *decostand* function of the *vegan* package in R (Jari Oksanen et al., 2018) in order to compare them to the relative abundances of the microscopic pollen concentrations. The RRA represents the proportion of reads for each taxon present in a sample out of the total reads for a sample. To visualize the taxonomic diversity and RRA distribution of *trn*L and nrITS2 in the three target taxa studied here, we used the *metabaR* package in R from Zinger et al. (2021).

To determine which marker performed best in quantifying pollen, the RRA values were regressed against relative abundance of pollen concentrations using least squares regression of the *lm* function in R base (Team, R. Core, 2013). Since this relationship has been shown to be taxon dependant (Bell et al., 2019), independent statistical analyses were performed for each of the three target taxa (*Alnus*, Cupressaceae and Urticaceae) and DNA marker combination (*trn*L or nrITS2). Another regression model was made using RRA values from any taxon in the entire dataset that had >5% relative abundance in the microscopic pollen concentration. For these regressions all molecular taxonomic assignments were adjusted to the maximum taxonomic resolution obtained using microscopic pollen identification (e.g., RRA values from all OTUs of Cupressaceae and Taxaceae and for *Alnus* all species were summed up). For the nrITS2 results, the RRA values were plotted for all species identified within the three target taxa.

Finally, to visualize the (dis)similarity of the pollen identifications in samples from the different pollen monitoring stations (South-east and West of the Netherlands) and the different seasons, the Bray-Curtis dissimilarity index was calculated using the RRA values of nrITS2 and *trn*L between each pair of samples using the *vegdist* function of the *vegan* package in R (Jari Oksanen et al., 2018). These values were ordinated using nonmetric multidimensional scaling (NMDS) and visualized with the *ordiplot* function in *vegan*, grouped per pollen monitoring site and per season. The statistical significance of the differences between these variables were tested using a permutational multivariate analysis of variance (perMANOVA) with 999 permutations, using the *adonis* function in *vegan*.

## 3 Results

### 3.1 Sequence run statistics

DNA was obtained from both the unmounted tapes and the microscopic slides that contained safranin and glycerin. For nrITS2, seven samples were discarded before sequencing because they did not yield any amplicons after two rounds of PCR. Illumina sequencing resulted in 7.5 M read pairs for nrITS2 and 8.6 M for *trn*L. After quality filtering and merging, 6.4 M reads remained for nrITS2 and 6.8 M for *trn*L. Respectively three and five samples were discarded because they had <3000 reads in all PCR replicates for nrITS2 and *trn*L. Forty-eight out of the 58 analysed samples were retained for nrITS2 and 53 for *trn*L (Table S1). Per sample read abundance was 52,775 ± 4671 for nrITS2 and 48,784 ± 4241 for *trn*L. While the average read abundance from samples that were mounted on microscopic slides was lower than those from unmounted tapes for both markers, they were sufficiently high for further analysis (40,368 and 40,742 reads for the mounted samples versus 59,257 and 48,325 reads for unmounted tapes for nrITS and *trn*L, respectively). Mean GC-content for nrITS2 amplicons was 58.4 ± 2.7% and for *trn*L 32.1 ± 7.3%.

### 3.2 Taxonomic resolution

**Fig. 2:**
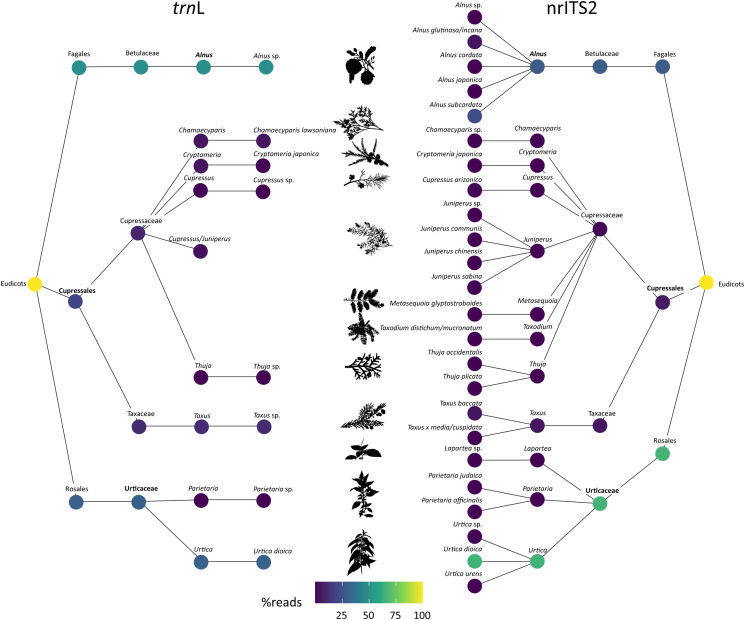
Taxonomic resolution for *Alnus*, Cupressaceae and Urticaceae achieved using *trn*L and nrITS2 metabarcoding of pollen grains collected with a Burkard sampler at two pollen monitoring sites in the Netherlands. Results from *trn*L are shown on the left side while nrITS2 is shown on the right. Colours of the circles represent the percentage of identified reads. The maximum taxonomic resolution achieved using microscopic pollen identification for the three target taxa is noted in bold.

Across all samples and markers, 56 plant families, 143 genera and 168 different plant species were identified (Figs. S1, S2; Tables S2 – S5). At the family level, all pollen identified by microscope was also found with metabarcoding. The total number of OTUs identified using nrITS2 was almost twice as high (191) as for *trn*L (98), and was also higher per sample for nrITS2 (14.4 ± 1.7) than for *trn*L (12.0 ± 1.0) (Table S1). For nrITS2, 80.1% of all OTUs could be identified to the species level, while this was 27.6% for *trn*L. Most species were uniquely identified using nrITS2 (141), while 15 species were only found using *trn*L and 12 were shared between the two markers (Fig. S3). Several families were identified using DNA that were not detected using the microscope. Families including Araliaceae, Equisetaceae, Myricaceae and Cornaceae were additionally identified by *trn*L, while Euphorbiaceae, Boraginaceae, Scrophulariaceae and Papaveraceae were additionally identified by nrITS2 (Fig. S1, S2). The Euphorbiaceae were represented by *Mercurialis annua* and *M. perennis*, species of potential allergenic importance (Ariano et al., 1993). Within the three target taxa *Alnus*, Cupressaceae and Urticaceae, nrITS2 identified four families, nine genera and 16 species while *trn*L identified four families, six genera and three species (Fig. 2). For *Alnus*, no taxa could be identified at species-level using *trn*L, while *Alnus cordata*, *A. japonica* and *A. subcordata* were identified by nrITS2. The latter two species are the parental species of the commonly planted artificial hybrid *Alnus* × *spaethii* (Spaeth's Alder). The native species *Alnus glutinosa* and *A. incana* could not be distinguished from each other using nrITS2. For Cupressaceae, *trn*L identified five genera and two species, with some genera that could not be distinguished (*Cupressus*/*Juniperus*). nrITS2 could distinguish eight genera within the Cupressaceae, with most identifications at the species level (nine). Within the Urticaceae, two taxa were distinguished by *trn*L (*Urtica dioica* and *Parietaria* sp.) while three genera (*Urtica, Parietaria* and *Laportea*) were distinguished using nrITS2, with two species in both *Urtica* and *Parietaria*.

### 3.3 Pollen quantification using metabarcoding

**Fig. 3:**
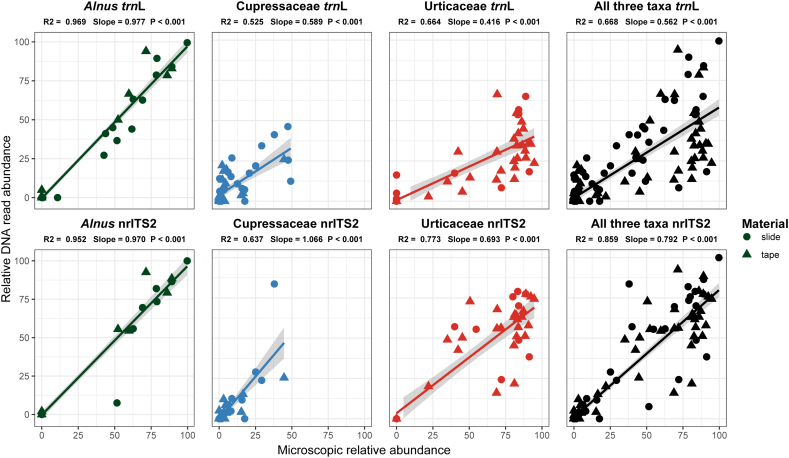
Correlations of microscopic pollen concentrations and sequencing read abundances. Regressions for *Alnus* sp., Cupressaceae, Urticaceae as well as all three combined are shown. The top panels show the results of *trn*L and the bottom panels nrITS2. Comparisons are at the maximum taxonomic levels these taxa can be identified with a microscope. Pollen concentrations were converted to relative abundances for comparison to DNA relative read abundances.

Highly significant positive relationships between the relative abundance of sequencing reads (RRA) and relative abundance of microscopically obtained pollen concentrations were found for all studied taxa using *trn*L and nrITS2 (*p* < 0.001 for all correlations; Fig. 3). For *Alnus* the highest correlation was found using *trn*L (R^2^ = 0.969) and nrITS2 (R^2^ = 0.952). For the other two target taxa a lower correlation was found using *trn*L (R^2^ = 0.525 and 0.664 for Cupressaceae and Urticaceae respectively) compared to nrITS2 (R^2^ = 0.637 and 0.773). The regression line slopes also had lower values using *trn*L (0.589 and 0.416 for Cupressaceae and Urticaceae respectively) compared to nrITS2 (1.066 and 0.693), while a slope of ~0.97 was found for *Alnus* in both markers. The relationships were not affected by the material used (microscopic slide or unmounted tape). When combining the RRA values from all taxa in the dataset with >5% relative abundance in the microscopic pollen concentrations, corresponding results were found with a R^2^ value of 0.620 and slope of 0.588 for all *trn*L data, while the R^2^ value was 0.821 for nrITS2, with a slope of 0.764 (Fig. S4).

### 3.4 Trends in plant species abundance

**Fig. 4:**
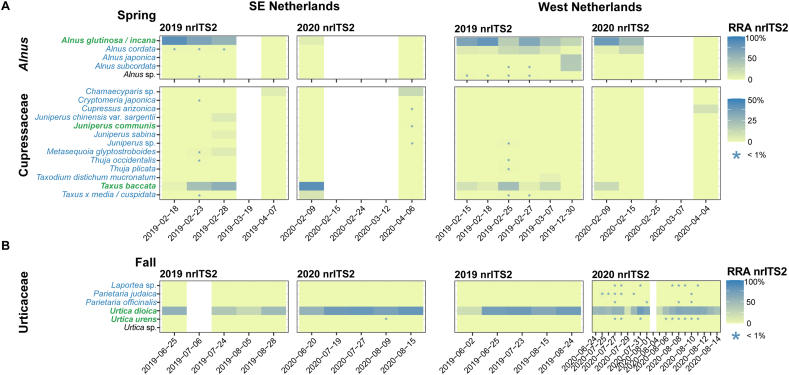
Relative nrITS2 molecular read abundance of species of *Alnus*, Cupressaceae in spring and Urticaceae in fall of the 2019 and 2020 seasons of two pollen monitoring sites in the Netherlands (West and South-east of the Netherlands). The x-axis represents the material collection dates (see Fig. 1). * presence at low relative abundance (< 1%). Taxa in green are native to the Netherlands, taxa in blue are either cultivated or introduced, and for taxa in black this is unknown. White bars indicate samples for which amplification failed. (For interpretation of the references to colour in this figure legend, the reader is referred to the web version of this article.)

Since nrITS2 results showed the highest taxonomic resolution and correlation between RRA and pollen concentrations, prevalence and presence of different plant species through time was only plotted for nrITS2 (Fig. 4). In spring, the genus *Alnus* was dominated by native *Alnus glutinosa* and *A. incana* for both studied pollen monitoring sites. DNA from pollen of non-native *Alnus cordata* was most abundantly identified in samples from late February 2019 in the West of the Netherlands (up to 26.6%), while only very low abundances of this species were found in the South-east of the Netherlands. Non-native *Alnus japonica* and *A. subcordata* were found in high abundance in the sample from late December 2019 in the West of the Netherlands. Cupressaceae show highly diverse species recovery in spring, but the pollen spectra are almost entirely dominated by *Taxus baccata* at both pollen monitoring stations. In April, for the South-east of the Netherlands non-native *Chamaecyparis* sp. was found, while this taxon was absent in the West of the Netherlands. Here, *Cupressus arizonica* was identified in the sample from April 2020. Native *Juniperus communis* was only found in very low abundance in April 2020 in the South-east of the Netherlands. In fall, Urticaceae pollen spectra are almost entirely dominated by *Urtica dioica* for both monitoring stations. *Urtica urens* was only found in low abundances in the fall of 2020 at both monitoring sites. Highly allergenic *Parietaria* species were detected in low abundances only in the West of the Netherlands in 2020. Finally, non-native *Laportea* was identified in the samples from the West of the Netherlands in 2020.

### 3.5 Comparison of monitoring sites and seasons

**Fig. 5:**
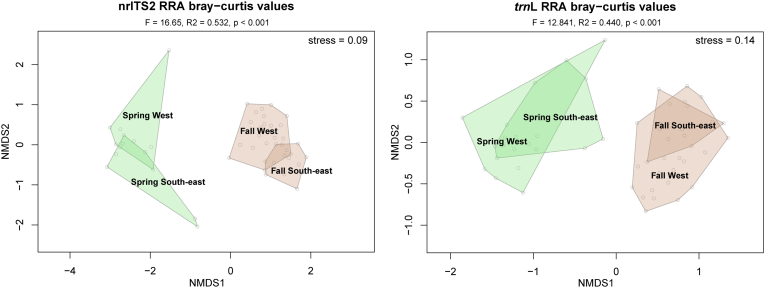
Two-dimensional NMDS plots on RRA-based Bray-Curtis dissimilarities of *trn*L and nrITS2 results from spring and fall at the West and South-east of the Netherlands. Polygons in green represent samples from spring while those in brown represent fall. (For interpretation of the references to colour in this figure legend, the reader is referred to the web version of this article.)

A perMANOVA of Bray-Curtis dissimilarities using RRA data of *trn*L and nrITS2 results showed significant discrimination between samples from spring and fall collected at the two Dutch pollen monitoring stations (*p* < 0.001 for both markers; Fig. 5). For nrITS2 a slightly higher R^2^ was found of 0.532 versus 0.440 for *trn*L. Spring and fall samples clearly fell within two separated groups for both markers, and within these groupings the samples from both stations also clustered together. For *trn*L a higher overlap was identified, especially between the samples from the fall for the two stations, while these were more separated in nrITS2.

## 4 Discussion

While previous studies have shown that DNA can be amplified from pollen collected by Hirst-type samplers (Banchi et al., 2020; Campbell et al., 2020; Kraaijeveld et al., 2015; Leontidou et al., 2017), our study presents the first successful amplification of DNA from pollen that have been stained and mounted on microscopic slides. This opens up opportunities of utilizing the vast historic resources of daily microscopic slides that have been collected for decades at pollen monitoring stations all over the world (see Buters et al., 2018 for an overview of pollen monitoring stations). DNA studies on historical pollen species dynamics in ambient air can potentially be reconstructed back in time using our methodology.

### 4.1 Molecular airborne pollen monitoring

Previous studies on aerobiological samples have mostly relied on plastid *rbc*L which has limitations in taxonomic resolution (mostly to the genus level) and relatively poor quantitative performances (Bell et al., 2017; Uetake et al., 2021). Although less samples were successfully amplified with nrITS2 than using *trn*L in our study (48 versus 53), the qualitative performance of nrITS2 was significantly better than plastid *trn*L with double the amount of OTUs and > 80% identified to the species level. Using *trn*L, several plant families were exclusively found that were also identified by microscopic pollen identification (Juncaceae and Pinaceae). However, these taxa were only present in <5% maximum relative abundance in the selected samples. In a recent study, Milla et al. (2021) found instead that *trn*L performed better than nrITS2 in Australian honey samples. However, as the authors indicate, DNA in these honey samples was degraded by long storage, causing the more stable and much shorter *trn*L P6 loop to be better preserved than nrITS2.

Our study adds to the growing body of evidence that nuclear markers are well suited for quantitative molecular pollen research (Banchi et al., 2020; Nunez et al., 2017; Richardson et al., 2021; Rowney et al., 2021). The correlation values for all taxa using *trn*L and nrITS2 in this study are very similar to those found in a recent study on bee-collected pollen quantification (Richardson et al., 2021). Here, at the genus level a relatively low correlation was found between *trn*L read proportions and microscopic proportions (R^2^ = 0.456, *p* < 0.001) while these values were much higher for nrITS2 (R^2^ = 0.846, *p* < 0.001). We focussed on samples with high abundances of pollen, as previous authors have identified that this correlation may be less significant for pollen types of low abundance (e.g. Rojo et al., 2019). Furthermore, and similar to previous studies, the relationships in our study were taxon dependent and showed differences in the correlation slope (e.g., Baksay et al., 2020; Bell et al., 2019). The slope for the genus *Alnus* was very close to 1 in *trn*L and nrITS2, indicating that for this taxon the relative abundance of reads is almost exactly equal to the relative abundance of pollen in microscopically obtained concentrations. For the family Urticaceae, however, a low slope value was found in the *trn*L results (0.416) and this underrepresentation of *trn*L RRA was also found for Urticaceae by Kraaijeveld et al. (2015). These species-specific differences may be a result of amplification bias, DNA isolation, preservation differences or copy number, as discussed by Bell et al. (2019). For plants, plastid and nuclear ribosomal ITS copy numbers per cell vary widely (Prokopowich et al., 2003). From our and previous quantification results (e.g. Bänsch et al., 2020; Richardson et al., 2021) it seems that plastid numbers per cell may be more variable than nuclear ribosomal copies, which may explain the better performance of nrITS2 versus *trn*L*.* Furthermore, plastid DNA is somewhat reduced in the paternal germ line, a feature that has led previous researchers to believe pollen did not contain any plastid DNA, although this has been disproven since (Bell et al., 2016b; Kraaijeveld et al., 2015). Previous studies have indicated that nrITS markers may be harder to amplify in plants as this marker has a relatively high GC content (Bell et al., 2016b; Mamedov et al., 2008; Richardson et al., 2019). This has led other researchers to find better quantification results using *trn*L compared to nrITS based on absolute read abundances (e.g., Baksay et al., 2020). However, in our study we find that very few taxa identified using a microscope were missed by nrITS2, and the ones that were missed (Juncaceae, Pinaceae) did not have a very high GC content but were more likely missed due to primer mismatches. When expected species contain high GC contents (>70%), amplification can be improved by adding DMSO additive to the PCR mix and/or lowering annealing temperatures (Varadharajan and Parani, 2021).

In this study, we included 58 aerobiological samples and only focused on three taxa. In future studies we aim to look at other stenopalynous taxa from other periods of the year where increased taxonomic resolution may be desirable, including e.g. Poaceae and Oleaceae, and to study the taxon-specific quantification results in more detail. Nevertheless, from the results of the three taxa, as well as from the results of all taxa present >5% in microscopic analysis (Fig. S4), we argue that nrITS2 should be the preferred marker of choice in molecular airborne pollen monitoring because of the highly increased taxonomic resolution and better semi-quantitative performance compared to *trn*L.

### 4.2 Pollen species dynamics

Using three case studies, we identified fine scale dynamics in species distribution patterns that could hitherto not be revealed. Within the allergenic genus *Alnus*, we find evidence that in late February a relatively large portion of the *Alnus* pollen is derived from non-native cultivated *Alnus cordata* (Italian alder)*,* while in December the peak is mainly caused by *Alnus* × *spaethii* (Fig. 4). The flowering periods of these alders prolong the alder hay fever season in the Netherlands. Traditionally, this was considered to last from February – early March (native *Alnus glutinosa* and *A. incana* flowering seasons), but *A. cordata* flowers from late February into early June (peak in April) and *A.* × *spaethii* from late December into early February (Duistermaat, 2020). These flowering periods correspond well with the dates in which we identified these species using nrITS2. *Alnus* × *spaethii* is of increasing interest to epidemiologists as it starts flowering significantly earlier than the native alders (Gehrig et al., 2015).

*Trn*L and nrITS2 could identify several genera within the Cupressaceae family including many that are not native to the Netherlands (e.g. *Cryptomeria, Chamaecyparis, Cupressus, Taxodium, Thuja*). Plants from these genera are popular ornamentals in gardens and city parks in the Netherlands. Some species are well-known causal agents of pollinosis in their native range (including *Cryptomeria japonica* in Japan and *Cupressus arizonica* in the Mediterranean; D'Amato et al., 2007; Yasueda et al., 1983). However, our results show that pollen from these species is relatively insignificant as compared to highly abundant *Taxus baccata* pollen (common yew; Fig. 4). Common yew is native to the Netherlands but is also often used as ornamental in hedges and gardens, which could explain its abundance in aerobiological samples. Even though yews are known to produce high amounts of pollen, their pollen is considered of low allergenic importance in Europe, as sensitization levels are very low (Puc et al., 2019). High cross-reactivity has been found, however, between Cupressaceae and Taxaceae (D'Amato et al., 2007).

For the Urticaceae pollen in fall, *Urtica dioica* plants are ubiquitous and highly abundant in the direct surroundings of both pollen monitoring stations, which explains the dominance of this species in the DNA results. Species of *Urtica* are of low allergenic relevance, but highly allergenic *Parietaria* spp. was additionally identified using both DNA markers. Although these genera can be distinguished using high resolution imaging and neural networks (Polling et al., 2021), they are not distinguishable using manual microscopic analysis. Species of *Parietaria* are one of the main causes of allergic rhinitis in the Mediterranean and they are currently undergoing a range expansion as a result of anthropogenic distribution and climate change (D'Amato et al., 2007; Fotiou et al., 2011). In the Netherlands *Parietaria* spp. are particularly abundant in the western, more urbanized part of the Netherlands (www.verspreidingsatlas.nl). This may explain why we only found DNA from *Parietaria* in the West of the Netherlands (Leiden) and none in the South-east (Helmond). One unexpected element in the nrITS2 results for Urticaceae was the presence of the genus *Laportea* in samples from the fall of 2020 in the West of the Netherlands, as species of this genus are native to the Americas, Africa and Australasia (Jiarui et al., 2003). *Laportea* is not native or in cultivation in the Netherlands, so either pollen arrived from long-distance transport or the sequences are the result of a sequencing error. The last option seems unlikely since the differences in the sequence to those of native *Urtica* and *Parietaria* were large (maximum identification of 80% to *Urtica dioica* while this was 95% for *Laportea*). Therefore, the first option seems more likely. Pollen has been found before to be able to travel long distances (de Weger et al., 2016), and even to the Arctic (Campbell et al., 1999). Unfortunately, the species of *Laportea* could not be distinguished due to <97% identity, but the closest match was *L. canadensis* (native to North America) with 95% identity.

### 4.3 Pollen monitoring sites and seasons

The two pollen monitoring sites could be distinguished based on the taxonomic compositions of fall and spring samples (Fig. 5). This was more clearly seen in the nrITS2 results than in *trn*L, likely because of the increased taxonomic resolution of nrITS2. The site-specific variation could be explained by native species that grow more or less exclusively in either the West of the Netherlands (e.g., *Spergularia media*, *Hippophae rhamnoides, Parietaria* spp.) versus the South-east of the Netherlands (e.g., *Juniperus communis*, *Quercus rubra* and *Mercurialis perennis*). Furthermore, several cultivated species were either only identified in the West of the Netherlands (e.g., *Phedimus* spp.*, Panicum virgatum, Alnus* × *spaethii*) or the South-east of the Netherlands (e.g., *Chamaecyparis* sp., *Cryptomeria japonica, Acer negundo*) indicating differences in the local environment surrounding the pollen monitoring sites. Lastly, some of the variance may be explained by a sampling effect, as more samples were used from the West of the Netherlands from the fall of 2020 (20) than from the South-east of the Netherlands (5). Nevertheless, both *trn*L and nrITS2 results could be used to infer statistically significant differences between the seasons and two pollen monitoring sites.

## 5 Conclusions

In this study we applied DNA metabarcoding on pollen from aerobiological samples collected at two monitoring stations in the Netherlands over a period of two consecutive years. Despite only focusing on two time periods and specifically targeting just three taxonomic groups (*Alnus*, Cupressaceae/Taxaceae and Urticaceae), the results show the huge added value of this technique. DNA was successfully amplified from microscopic pollen slides as well as unmounted Burkard-collected tapes, with no detected difference in the results. Where manual pollen identification detected 23 plant genera and 22 families, DNA metabarcoding using the two markers *trn*L and nrITS2 resulted in a total of 168 species from 143 genera and 56 plant families. Both markers identified taxa that were not detected using microscopic pollen identifications, including several of allergenic importance (*Mercurialis* spp., *Parietaria* spp.). Important to note is that besides DNA from pollen, the source of the plant-DNA in aerobiological samples can also be plant debris. nrITS2 showed a much higher number of uniquely identified species (141) than *trn*L (15), with only 12 species found by both markers. Moreover, regressing the relative DNA read abundances for the three target taxa against the relative abundances of microscopic pollen concentrations, a consistently higher positive correlation was identified for nrITS2 than for *trn*L. This result was corroborated when looking at all taxa in the dataset with >5% relative abundance in the microscopic pollen concentrations. Significant differences were identified between the correlation slopes of the three target taxa, and future studies should focus on targeting more species.

Using the nrITS2 results, it was shown that *Alnus* in spring is dominated by native *Alnus glutinosa/incana*, whereas a significant amount of the non-native cultivated hybrid *Alnus* × *spaethii* is present in late December. Cupressaceae and Urticaceae at both monitoring stations were dominated by low-allergenic *Taxus baccata* and *Urtica dioica*, respectively. Lastly, the nrITS2 results allowed finer-scale spatiotemporal patterns to be distinguished between the pollen monitoring stations than using *trn*L. In conclusion, this study provides relevant insights into aerobiological species dynamics and shows that semi-quantitative molecular pollen monitoring is feasible at the species level, particularly using nrITS2.

## Funding information

This work was financially supported by the European Union‘s Horizon 2020 research and innovation programme under H2020 MSCA-ITN-ETN grant agreement No 765000 Plant.ID.

## CRediT authorship contribution statement

**Marcel Polling:** Conceptualization, Investigation, Methodology, Visualization, Formal analysis, Software, Writing – original draft. **Melati Sin:** Investigation, Formal analysis. **Letty A. de Weger:** Validation, Resources, Investigation, Writing – review & editing. **Arjen Speksnijder:** Methodology, Software, Writing – review & editing. **Mieke J.F. Koenders:** Resources, Writing – review & editing. **Hugo de Boer:** Funding acquisition, Supervision, Writing – review & editing. **Barbara Gravendeel:** Supervision, Project administration, Funding acquisition, Writing – review & editing.

## Declaration of competing interest

The authors declare no conflict of interest.

## Appendix A Supplementary data

Supplementary figures Image 1

Supplementary tables Image 2
