# Supplementary material for: DNA metabarcoding using nrITS2 provides highly qualitative and quantitative results for airborne pollen monitoring
Source: Sci Total Environ. 2022 Feb 1;806:150468. doi: 10.1016/j.scitotenv.2021.150468 (PMC8651626; doi:10.1016/j.scitotenv.2021.150468)
Supplement: Supplementary file 1 — Supplementary figures [file mmc1.docx]

**DNA metabarcoding using nrITS2 provides highly qualitative and quantitative results for airborne pollen monitoring**

**Marcel Polling^1,2^, Melati Sin^1^, Letty A. de Weger^3^, Arjen G.C.L. Speksnijder^1,4^, Mieke J.F. Koenders^5^, Hugo de Boer^1,2^, Barbara Gravendeel^1,6^**

^1^Naturalis Biodiversity Center, Leiden, The Netherlands
^2^Natural History Museum, University of Oslo, Norway
^3^Department of Pulmonology, Leiden University Medical Center, Leiden, The Netherlands
^4^Leiden University of Applied Sciences, Leiden, The Netherlands
^5^Clinical Chemistry, Elkerliek Hospital, Helmond, The Netherlands
^6^Radboud Institute for Biological and Environmental Sciences, Nijmegen, The Netherlands

***Supplementary Information (1/2)***

Contents

Figure S1. All nrITS2 results 1

Figure S2. All *trn*L results 3

Figure S3. Venn diagrams all data 4

Figure S4. RRA Correlations 5

# Figure S1. All nrITS2 results

*Taxa in red have been filtered out, either because it wasn’t an eudicot, or because it was interpreted as contamination. Taxa in bold were uniquely found in the DNA results of nrITS2.*


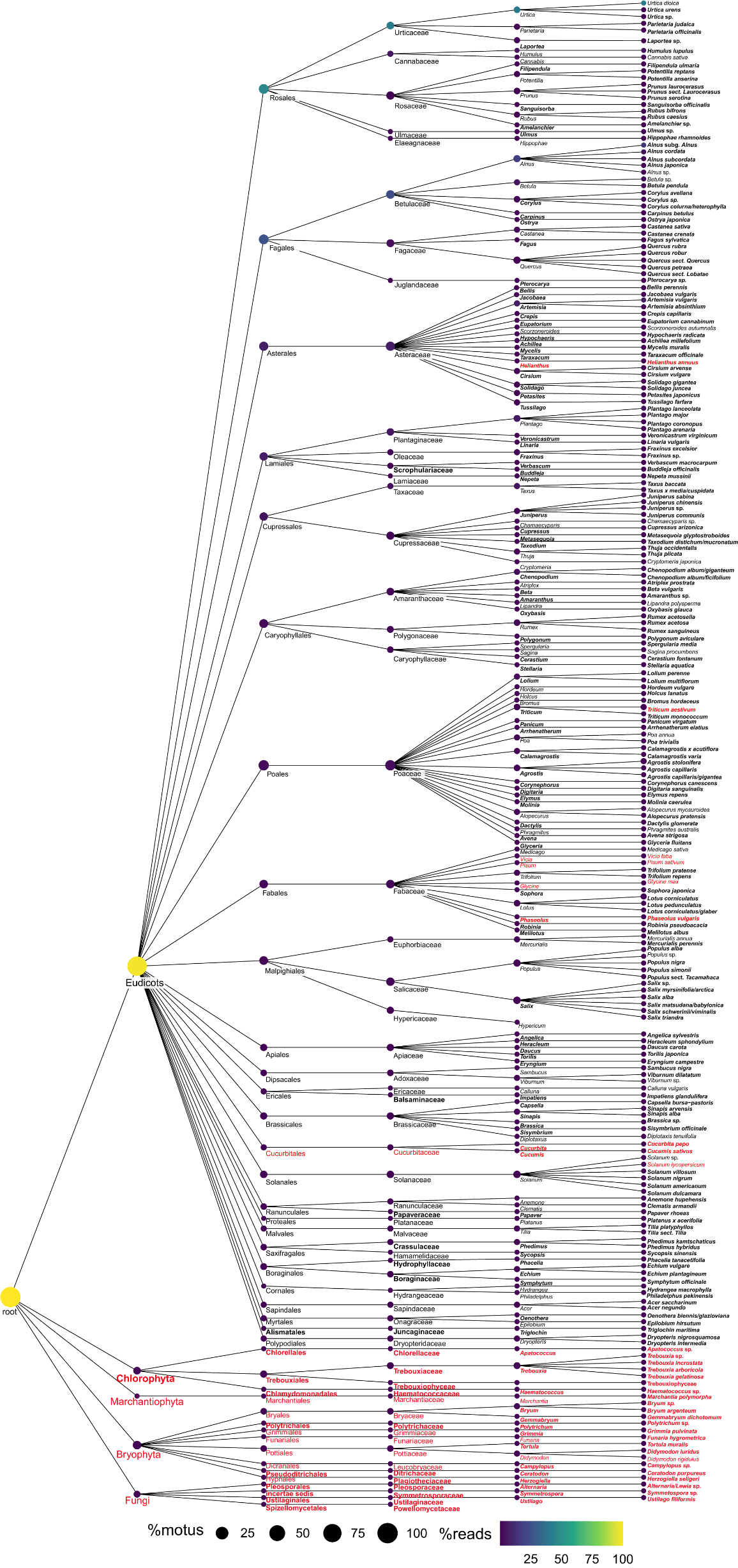


# Figure S2. All *trn*L results

*Taxa in red have been filtered out, either because it wasn’t an eudicot, or because it was interpreted as contamination. Taxa in bold were uniquely found in the DNA results of trn*L*.*


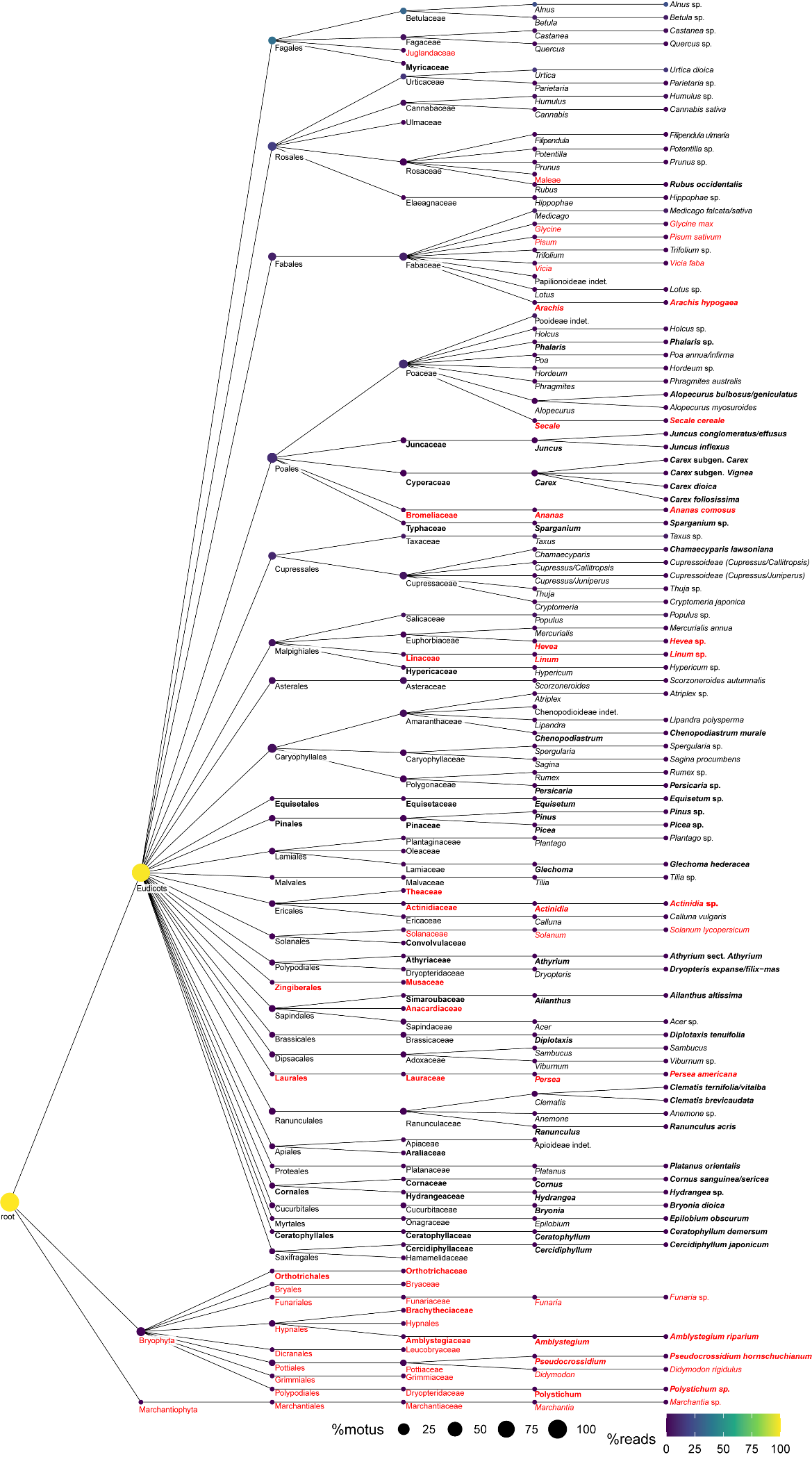


# Figure S3. Venn diagrams all data

**a.**


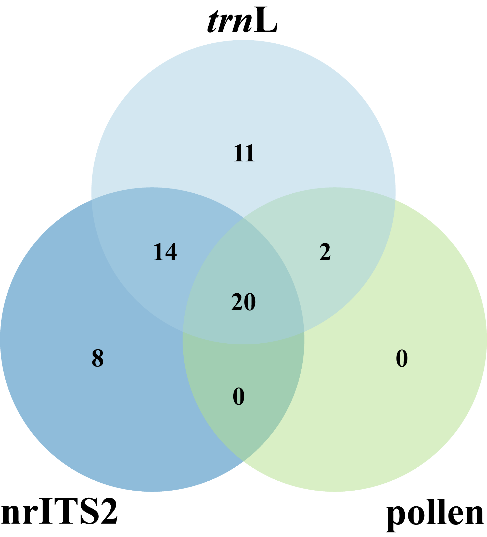

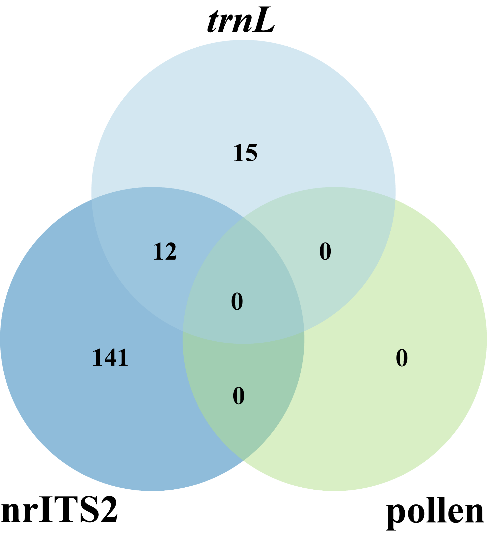


**b.**


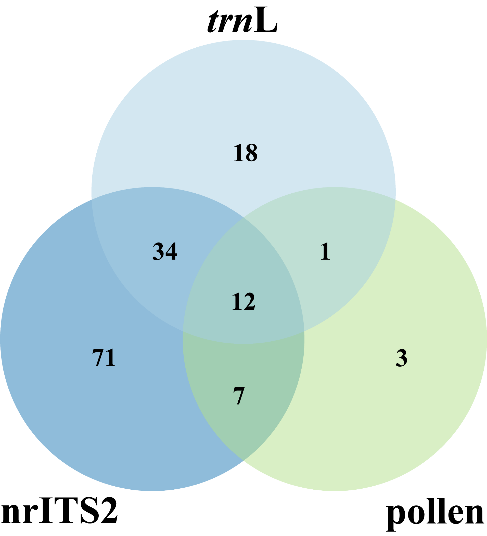


**c.**

Figure S3. Venn diagrams of all recovered taxa at different taxonomic levels a) family, b) genus and c) species level

#
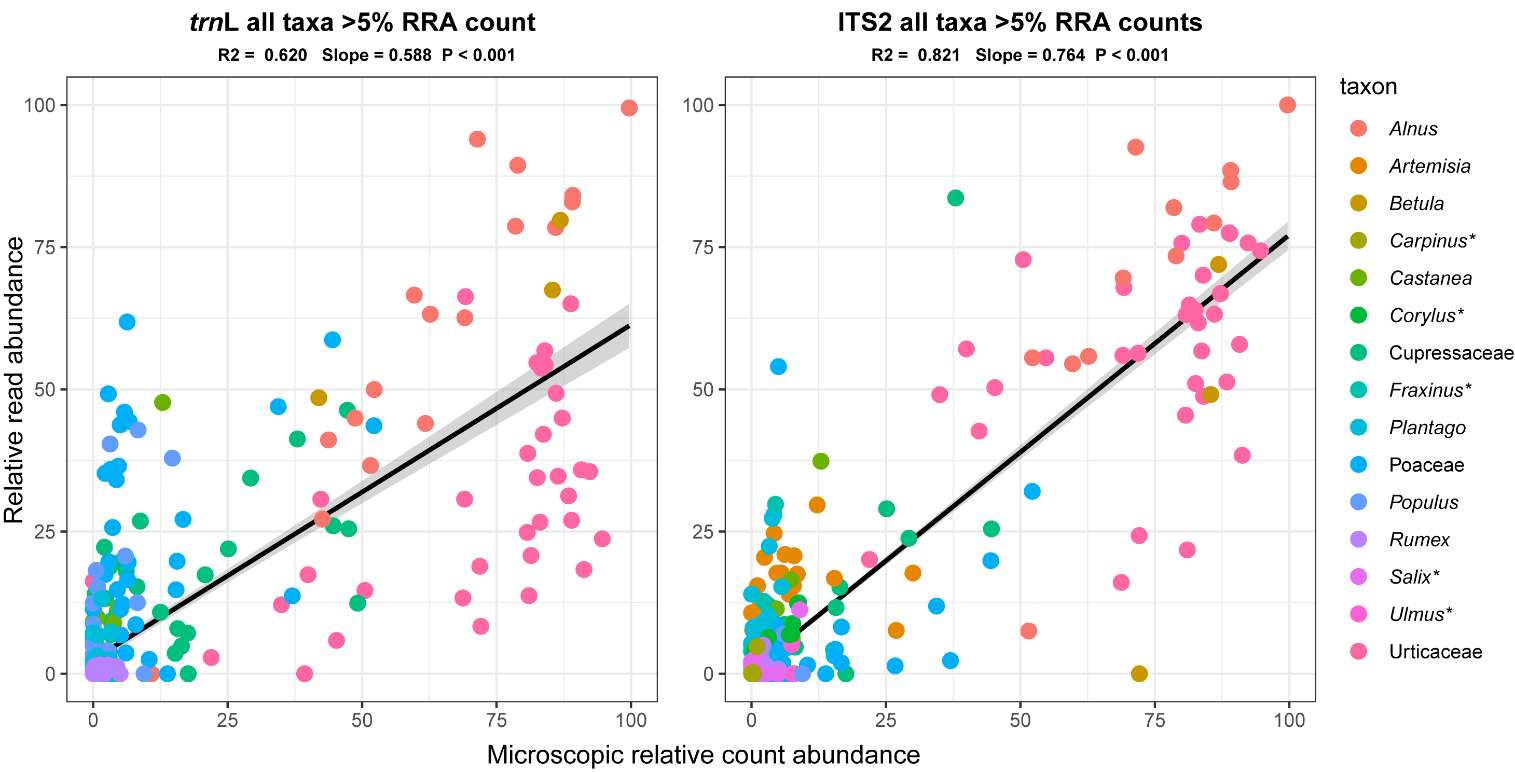
Figure S4. RRA Correlations

Figure S4. Molecular relative read abundance regressed against relative abundance of microscopic pollen counts for all taxa recovered using trnL and nrITS2 in the 58 studied aerobiological samples. Taxa are indicated using unique colors, showing only those that were present in >5% relative abundance in the microscopic pollen counts. Comparisons are performed at the maximum taxonomic level that can be achieved using microscopic pollen identification. Taxa denoted with a * were only identified using nrITS2.
